# Semaphorin 4B promotes tumor progression and associates with immune infiltrates in lung adenocarcinoma

**DOI:** 10.1186/s12885-022-09696-w

**Published:** 2022-06-08

**Authors:** Jun Jiang, Yuan Lu, Fang Zhang, Tao Pan, Zhipei Zhang, Yi Wan, Xinling Ren, Rui Zhang

**Affiliations:** 1 Department of Health Service, Base of Health Service, Fourth Military Medical University, Xi’an, China; 2 Department of Respiratory and Critical Care Medicine, Zhongda Hospital, Southeast University, Nanjing, China; 3 Department of Respiratory and Critical Care Medicine, Xijing Hospital, Fourth Military Medical University, Xi’an, China; 4 Translational Medicine Center, The Second Affiliated Hospital of Guangzhou Medical University, Guangzhou, China; 5 Department of Thoracic Surgery, Tangdu Hospital, Fourth Military Medical University, Xi’an, China; 6 Department of Pulmonary Medicine, Shenzhen General Hospital, Shenzhen University, Shenzhen, 518055 Guangdong China; 7 State Key Laboratory of Cancer Biology, Department of Immunology, Fourth Military Medical University, Xi’an, 710032 Shaanxi China

**Keywords:** SEMA4B, Lung adenocarcinoma, Immune infiltration, Bioinformatics, Prognosis

## Abstract

**Background:**

Semaphorins have been found to play important roles in multiple malignancy-related processes. However, the role of Semaphorin 4B (SEMA4B) in lung cancer remains unclear. Here, we aimed to explore the biological functions of SEMA4B in through bioinformatic analysis, in vitro and in vivo assays. In the present study, the possible mechanism by which SEMA4B affected the tumor growth and microenvironment of lung adenocarcinoma (LUAD) were investigated.

**Methods:**

The expression of SEMA4B in LUAD was analyzed by bioinformatic analysis and verified by the immunohistochemistry staining. The prognostic value of SEMA4B in LUAD was investigated using the Kaplan-Meier survival and Cox’s regression model. After silencing SEMA4B expression, the functions of SEMA4B in LUAD cells were investigated by in vitro experiments, including CCK-8 and plate clone formation. And the effect of SEMA4B on tumor growth and immune infiltration was explored in C57BL/6 mice tumor-bearing models.

**Results:**

SEMA4B expression was upregulated in LUAD tissues and correlated with later pathological stages and poor prognosis of LUAD patients. Further study found that SEMA4B silencing suppressed the proliferation of lung cancer cells both in vitro and in vivo. Bioinformatic analysis showed that SEMA4B expression was correlated with the increased infiltration of myeloid-derived suppressor cells (MDSCs), T-regs and the decreased infiltration of CD8^+^ T cell in LUAD. Importantly, in vivo study verified that the infiltration of T-regs and MDSCs in tumor microenvironment (TME) of Xenograft tissues was decreased after SEMA4B silencing.

**Conclusions:**

These findings demonstrated SEMA4B might play an oncogenic role in LUAD progression, and be a promising therapeutic target for lung cancer.

**Supplementary Information:**

The online version contains supplementary material available at 10.1186/s12885-022-09696-w.

## Background

Lung cancer is the most common cause of cancer-related deaths worldwide [1]. Five-year survival rate of lung cancer varies from 4 to 17%. Non-small cell lung cancer (NSCLC) account for about 80 to 85% of total lung cancer cases, and lung adenocarcinoma (LUAD) is the most common subtype of NSCLC [2]. For patients with early-stage NSCLC, adjuvant chemotherapy and surgery are the standard treatment strategies, whereas molecular targeted therapy, immunotherapy, chemotherapy and radiotherapy are advised for patients with advanced-stage diseases [3]. Although small molecule inhibitors targeting EGFR or ALK have shifted the paradigm for the treatment of lung cancer, the prognosis of patients initially harboring sensitive mutations remains unsatisfactory because of acquired drug resistance [4]. Moreover, patients without sensitive mutations could barely benefit from current molecular targeted therapy, and treatment options for them were usually restricted to cytotoxic chemotherapy in the past. Recently, the beneficial population of immune checkpoints inhibitors (ICIs) targeting PD-1, PD-L1 or CTLA-4 have dramatically increased, who experience longer overall survival (OS) than patients treated with chemo-monotherapy [5]. However, the fact is that only a small proportion of NSCLC patients respond to ICIs monotherapy, and not all responders continue to respond indefinitely [6]. Therefore, it is clinical importance to understand the underlying mechanism of drug resistance to ICIs and identify patients who may benefit from ICIs treatment.

Semaphorins (SEMAs) constitute a large family of secreted, transmembrane and cell surface-attached proteins that are involved in regulating various cell-to-cell communications [7]. There are more than 20 kinds of Semaphorins which can be divided into 3–7 categories in vertebrates. Class 3 Semaphorins are secreted proteins, classes 4 to 5 Semaphorins are membrane-bound proteins, and SEMA7A is the only GPI-anchored protein [8, 9]. Misexpression of Semaphorins disturbs various tissue and organ functions [10, 11]. Moreover, recent studies have indicated that Semaphorins play important roles in cancer progression by remodeling tumor parenchyma, facilitating angiogenesis, and modulating immuno-survillence. Firstly, Semaphorins, like SEMA3A in breast cancer, could directly affect growth, motility and metastasis of tumor cells by binding with their co-receptors NRP-1 [12]. Secondly, studies have shown that tumor angiogenesis could be facilitated due to upregulation of pro-angiogenic Semaphorins and simultaneous downregulation of anti-angiogenic Semaphorins [12, 13]. Thirdly, Semaphorins such as SEMA4D have been linked with the regulation of immune cell infiltration in NSCLC. Anti-SEMA4D used in patients facing progression after ICIs therapy received favorable results with a disease control rate of 81% [14].

SEMA4B, belonging to the Class SEMA4, is a transmembrane homodimer glycoprotein. Lots of SEMA4 family members have been implicated in the formation and progression of tumors due to. For example, SEMA4C promotes tumor growth by serving as an attractant recruiter of tumor associated macrophages (TAMs) [15]. SEMA4D might be related to dysfunction or exhaustion of immune cells in NSCLC [16]. The results from RNA-seq and flow cytometry using biopsy specimens found SEMA4B commonly up-regulated in subpopulations of pro-metastatic B cells in clear cell renal cell carcinoma [17]. Importantly, bioinformatic studies indicated SEMA4B may be an immune-related biomarker that could improving the prediction of prognosis in lung cancer [18, 19]. Although aberrant SEMA4B activity has been observed in multiple types of malignancies, the underlying roles of SEMA4B in lung cancer are still uncertain [20].

In our study, we collected surgical specimens from LUAD patients and found that SEMA4B is significantly upregulated. Then, we systematically evaluated the significance of SEMA4B in LUAD by analyzing RNA-seq data from The Cancer Genome Atlas (TCGA) database, along with bioinformatic analysis including differentially expressed genes (DEGs) analysis [21], TIMER 2.0 [22], gene ontology (GO) term analysis, Kyoto Encyclopedia of Genes and Genomes (KEGG) pathway analysis [23], gene set enrichment analysis (GSEA), and Kaplan-Meier survival analysis. We also proved SEMA4B could promote tumor proliferation in Lewis lung cancer (LLC) both in vitro and in vivo. Furthermore, SEMA4B might mediate immune evasion of LUAD by increasing recruitment of immunosuppressive cells, such as regulatory T cells (Tregs) and myeloid-derived suppressor cells (MDSCs).

## Results

### The SEMA4B expression is elevated in multiple types of solid tumors including LUAD

**Fig. 1:**
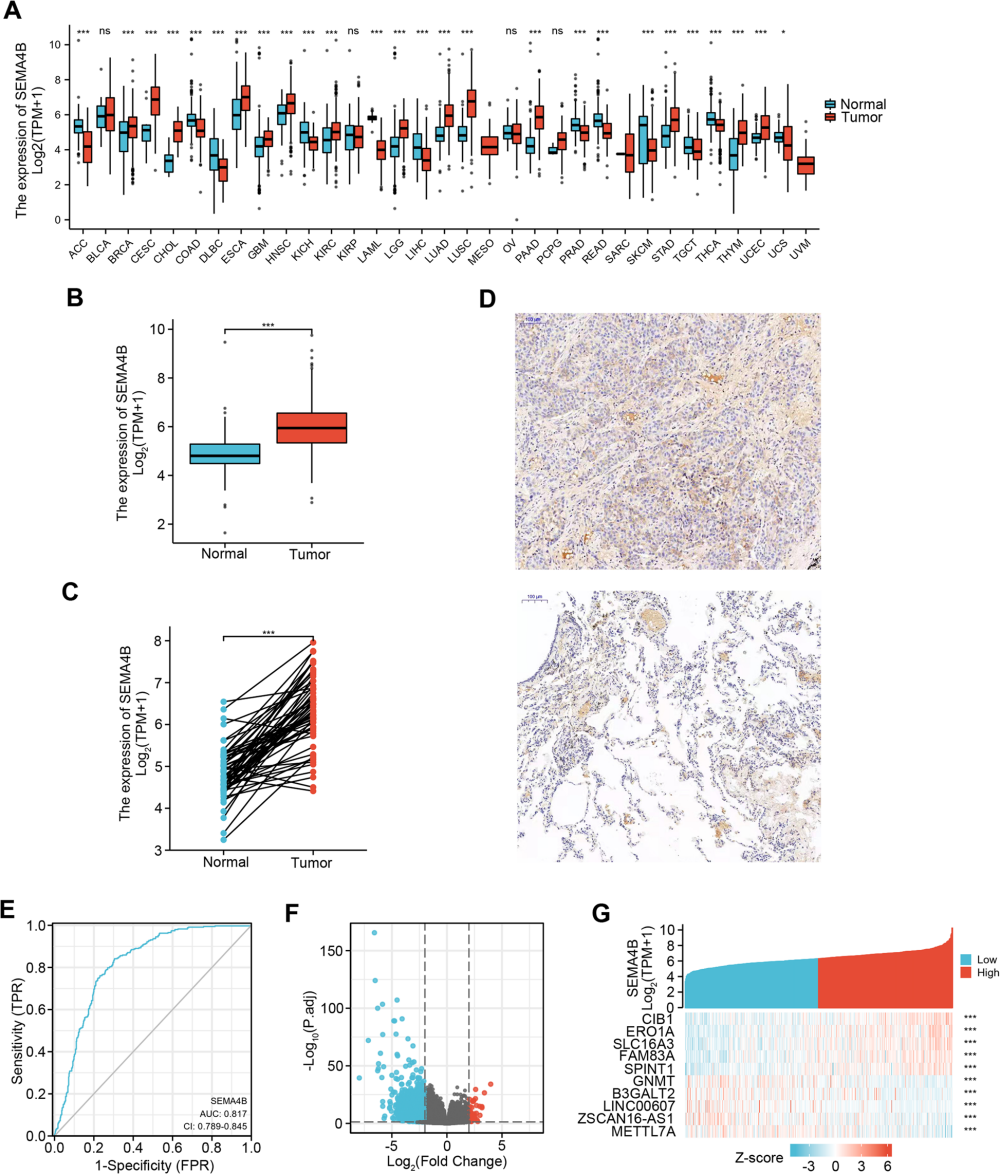
Differential expression of SEMA4B in various malignancies and SEMA4B-related differentially expressed genes (DEGs). **A** Upregulation or downregulation of SEMA4B in different malignancies compared with normal tissues in the TCGA and GTEx database. **B** Differential expression of SEMA4B in unpaired samples of LUAD. **C** Differential expression of SEMA4B in paired samples of LUAD. **D** The expression of SEMA4B in LUAD (up) and normal (down) tissues (100×) (**E**) A ROC curve about the value of SEMA4B to identify LUAD. **E**, **F** Volcano plots of the DEGs and heat map of the SEMA4B-related DEGs

To provide a comprehensive evaluation of SEMA4B expression in malignances, we compared the expression of SEMA4B across 33 TCGA cancer types. We found that SEMA4B was significantly upregulated in most types of cancers, including LUAD (Fig. 1A). We then compared SEMA4B expression in 515 LUAD samples with 347 normal samples. LUAD tumors had significantly higher SEMA4B (*P* = 0.000) (Fig. 1B). SEMA4B expression in 57 LUAD samples was also significantly upregulated as oppose to matched paracancerous samples (*P* = 0.000), suggesting that high SEMA4B-expression may play an important role in driving LUAD tumorgenesis (Fig. 1C). To verify the expression of SEMA4B in LUAD samples, we performed and screened IHC staining. As shown in Fig. 1D, SEMA4B was highly expressed in LUAD compared with normal tissue. Importantly, there were more CD11b and Foxp3 staining in SEMA4B positive LUAD samples as shown in sFig. 3, which means that SEMA4B expression might associated with MDSC (CD11b^+^) and Treg (Foxp3^+^) infiltration.

As shown in Fig. 1E, ROC curve was produced to investigate the diagnostic accuracy of SEMA4B and its capability to distinguish LUAD tissues from normal lung tissues. Its potential as a diagnostic biomarker in LUAD was confirmed, as the area under the ROC curve (AUC) was 0.817 (95% confidence interval [CI], 0.789–0.845), representing a moderate ability to discriminate LUAD from normal tissues (Fig. 1E). The optimal cutoff value was 5.499, which yielded a sensitivity (true positive rate) of 84.1% and a specificity of 69.5% for predicting the presence of LUAD.

### Identification of DEGs in LUAD

Comparing the gene expression profiles obtained for LUAD vs. normal samples, 2493 genes were significantly differentially expressed by more than 2-fold (*p*.adj<0.05) in LUAD with 33 upregulated and 2460 downregulated (Fig. 1F). Further analysis found that top 5 DEGs associated with SEMA4B-high are CIB1, ERO1A, FAM83A and SPINT1, whereas top 5 DEGs associated with SEMA4B-low are GNMT, B3GALT2, LINC00607, ZSCAN16-AS1 and METTL7A (Fig. 1G).

### High SEMA4B expression is significantly associated with clinical progression and poor prognosis in LUAD patients

**Fig. 2:**
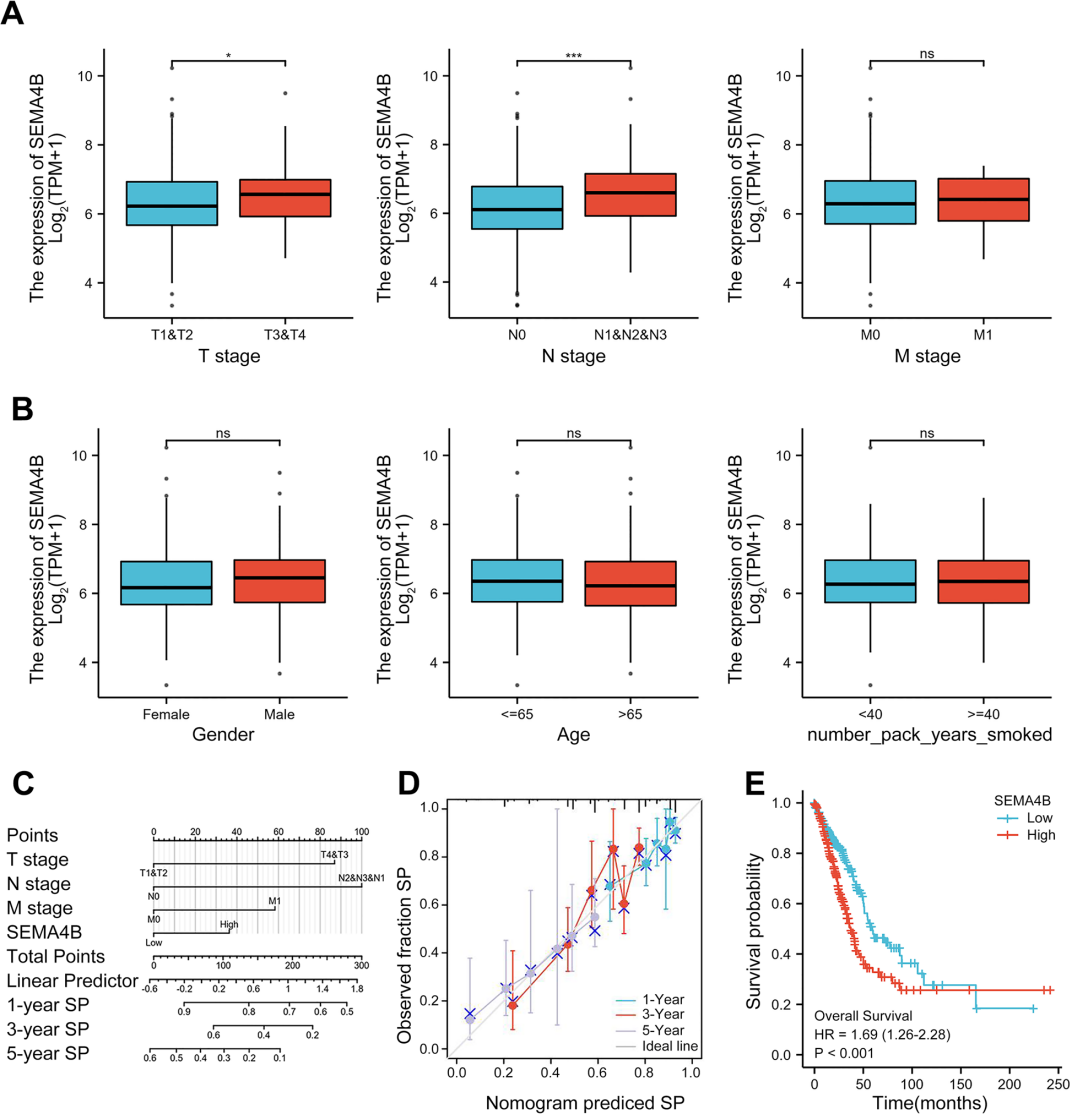
The association between SEMA4B expression and clinical characteristics, including (**A**) TNM stage, (**B**) gender, age and smoke status. (**C**) A nomogram for predicting LUAD patients the probability of 1-, 3-, and 5- year OS. SP, Survival Probability. (**D**) Calibration plots of the nomogram. (**E**) Survival curves of OS between high and low SEMA4B expression in LUAD

We analyzed 535 LUAD samples from TCGA to explore the relation between clinical characteristics and SEMA4B expression. As shown in Table 1, overexpressed SEMA4B was significantly correlated with pathologic stage (*p* <  0.001), T stage (*P* = 0.004), N stage (*p* <  0.001), gender (*p* = 0.006), and OS (*p* = 0.016). SEMA4B expression has no relation with other clinic-pathological characteristics. The univariate analysis with Logistic regression show that SEMA4B upregulation was significantly associated with advanced clinical stage (OR = 1.732, stage III/IV vs. stage I/II, *p* = 0.013), including T stage (OR = 1.981,T3/4 vs. T1/2, *p* = 0.011) and N stage (OR = 2.135, N1/2/3 vs. N0, *p*<0.001), but not M stage (M1 vs. M0, *p* = 0.608), which indicated that SEMA4B might promote LUAD cell proliferation and lymphatic metastasis (Fig. 2A and Table 2). Meanwhile, no significant difference was found in expression of SEMA4B between gender, age and smoking time (Fig. 2B).

**Table 1:** The association between SEMA4B expression and clinicopathological variables

Characteristic	Low expression of SEMA4B	High expression of SEMA4B	p
n	267	268	
T stage, n (%)			0.004
T1	104 (19.5%)	71 (13.3%)	
T2	137 (25.8%)	152 (28.6%)	
T3	16 (3%)	33 (6.2%)	
T4	8 (1.5%)	11 (2.1%)	
N stage, n (%)			< 0.001
N0	193 (37.2%)	155 (29.9%)	
N1	36 (6.9%)	59 (11.4%)	
N2	27 (5.2%)	47 (9.1%)	
N3	0 (0%)	2 (0.4%)	
M stage, n (%)			0.759
M0	178 (46.1%)	183 (47.4%)	
M1	11 (2.8%)	14 (3.6%)	
Pathologic stage, n (%)			< 0.001
Stage I	172 (32.6%)	122 (23.1%)	
Stage II	47 (8.9%)	76 (14.4%)	
Stage III	31 (5.9%)	53 (10.1%)	
Stage IV	12 (2.3%)	14 (2.7%)	
Gender, n (%)			0.006
Female	159 (29.7%)	127 (23.7%)	
Male	108 (20.2%)	141 (26.4%)	
Race, n (%)			0.937
Asian	4 (0.9%)	3 (0.6%)	
Black or African American	27 (5.8%)	28 (6%)	
White	209 (44.7%)	197 (42.1%)	
Age, n (%)			0.427
<=65	122 (23.6%)	133 (25.8%)	
> 65	135 (26.2%)	126 (24.4%)	
Smoker, n (%)			1.000
No	37 (7.1%)	38 (7.3%)	
Yes	221 (42.4%)	225 (43.2%)	
OS event, n (%)			0.016
Alive	185 (34.6%)	158 (29.5%)	
Dead	82 (15.3%)	110 (20.6%)	
PFI event, n (%)			0.765
Alive	152 (28.4%)	157 (29.3%)	
Dead	115 (21.5%)	111 (20.7%)	
Age, meidan (IQR)	67 (60, 72)	65 (57, 72)	0.136

**Table 2:** SEMA4B expression association with clinical pathological characteristics (logistic regression)

Characteristics	Total(N)	Odds Ratio (OR)	*P* value
T stage (T3&T4 vs. T1&T2)	532	1.981 (1.177–3.409)	0.011
N stage (N1&N2&N3 vs. N0)	519	2.135 (1.470–3.119)	< 0.001
M stage (M1 vs. M0)	386	1.238 (0.549–2.861)	0.608
Pathologic stage (Stage III&IV vs. Stage I&II)	527	1.723 (1.127–2.659)	0.013

Our study revealed that high SEMA4B expression was associated with shorter OS [hazard ratio (HR):1.325; 95% confidence interval (CI): 1.151–1.526; *P*<0.001] as shown in Table 3. Furthermore, SEMA4B expression was an independent risk factor for the prognosis (OS) of LUAD patients as demonstrated with multivariate Cox regression analysis, which significantly contributed to mortality [HR:1.224; 95% CI: 1.030–1.455; *p* = 0.022]. T and N stage were independently prognostic for survival as well.

**Table 3:** Univariate regression and multivariate survival method (Overall Survival) of prognostic covariates in patients with LUAD

Characteristics	Total(N)	Univariate analysis		Multivariate analysis	
		Hazard ratio (95% CI)	*P* value	Hazard ratio (95% CI)	*P* value
Pathologic stage	518				
Stage I& II	411	Reference			
Stage III& IV	107	2.664 (1.960–3.621)	< 0.001	2.414 (1.765–3.303)	< 0.001
T stage	523				
T1&T2	457	Reference			
T3&T4	66	2.317 (1.591–3.375)	< 0.001	2.016 (1.321–3.076)	0.001
N stage	510				
N0&N1	437	Reference			
N2&N3	73	2.321 (1.631–3.303)	< 0.001	1.958 (1.294–2.962)	0.001
M stage	377				
M0	352	Reference			
M1	25	2.136 (1.248–3.653)	0.006	1.818 (1.023–3.231)	0.042
SEMA4B	526	1.325 (1.151–1.526)	< 0.001	1.224 (1.030–1.455)	0.022

The nomogram for estimating 1-, 3-, 5- year survival probabilities was built and validated on the base of Cox regression analysis as shown in Fig. 2C. For example, a patient with high SEMA4B expression (36.5), late T stage (87 points) and distant metastasis (57.5 points) received a total point score of 181. The probabilities of 1-, 3-, 5- year survival was about 72, 33, and 10%, respectively. The C-index was 0.679 (0.653–0.704), which suggested that the model had moderate prediction accuracy. The calibration plots of the nomogram showed that the agreement between predicted and observed OS was optimal (close to 45-degree ideal line) (Fig. 2D). Moreover, high SEMA4B expression was associated with a much shorter survival. Median OS (HR = 1.69, CI 1.26–2.28, *p* = 0.000) of patients with high SEMA4B expression was 37.2 months compare to 59.3 months in patients with low SEMA4B expression (Fig. 2E).

### SEMA4B promotes proliferation of lung cancer cells both in vivo and in vitro

**Fig. 3:**
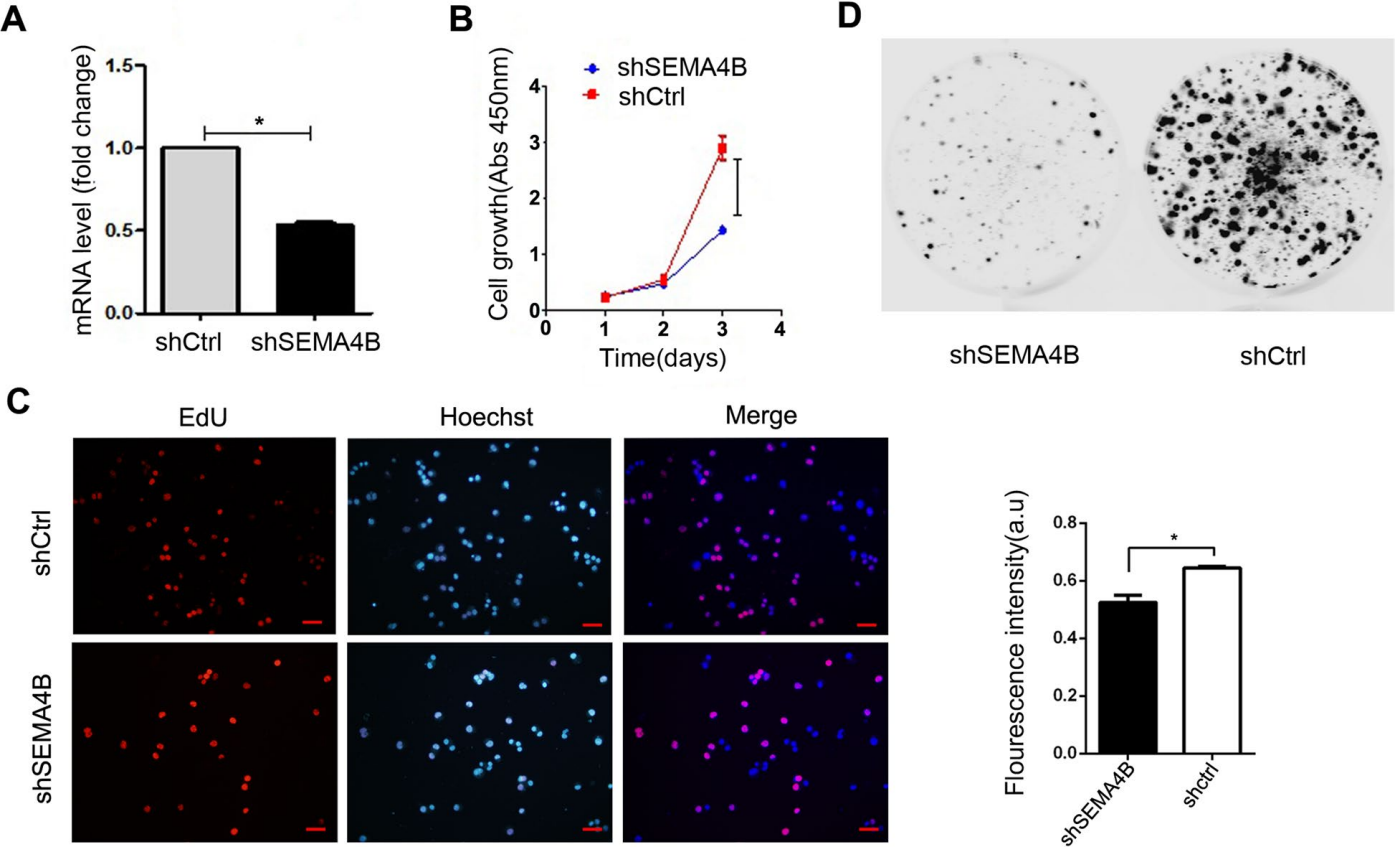
Effects of SEMA4B silencing on the proliferation of lewis lung cancer cells. **A** mRNA levels of SEMA4B after transfection of shSEMA4B and shCtrl. **B**-**C** CCK8 and EDU proliferation assay. **D** Plate clone formation assay. Error bars, means ± SD, *p < 0.05

To understand the role of SEMA4B in LUAD cell proliferation, we first constructed a recombinant virus in which the SEMA4B gene was fused with the reporter luciferase gene, and then established LLC clones stably expressing luciferase. We next assessed the effect of SEMA4B knockdown by transfection of cells with control siRNA or SEMA4B siRNA sequence shSEMA4B. SEMA4B knockdown was confirmed with qPCR for SEMA4B at 72 h (Fig. 3A). The CCK8 and Edu assay (Fig. 3B-C) and plate cloning formation assay (Fig. 3D) shown that SEMA4B knockdown by transfection of cells with siRNA could inhibit cell proliferation and reduce colony formation of lung cancer cells in vitro.

**Fig. 4:**
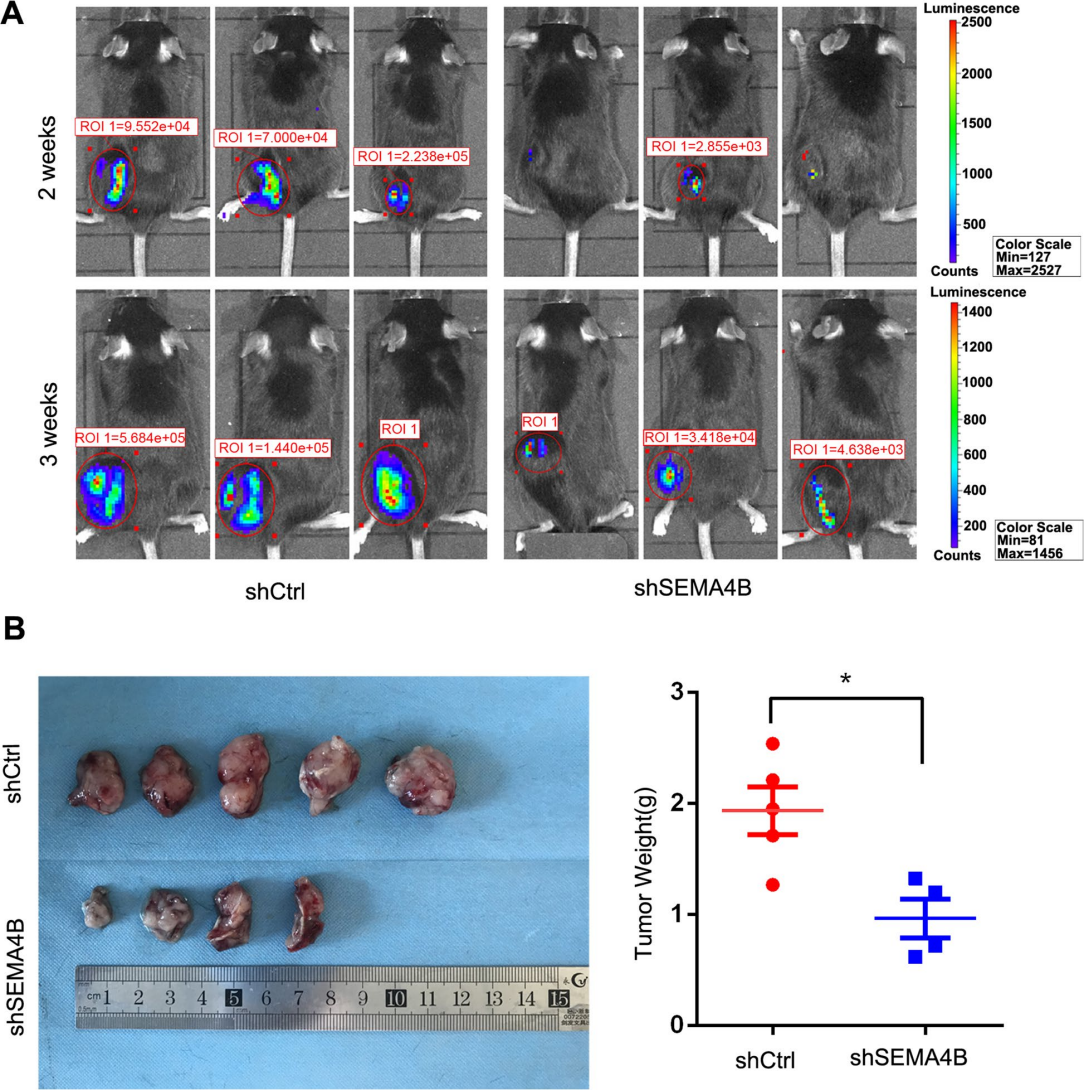
Effects of SEMA4B silencing on the growth of xenotransplanted tumors. **A** The bioluminescence intensity of C57 mice (*n* = 6). **B** Comparison of tumor weights in shSEMA4B and shCtrl group. Error bars, means ± SD, *p < 0.05

To investigate the fate of SEMA4B in vivo, we injected 2 × 10^6^ shSEMA4B/shCtrl-expressing bioluminescent LLC tumor cells subcutaneously to thigh of C57BL/6 mice to monitor tumor growth. Tumor growth was measured by living imaging system at the end of the second and third week after tumor inoculation. By three weeks, all mice of the shSEMA4B group showed significantly impairing tumor development represented by bioluminescence intensity. In contrast, the shCtrl group demonstrated continuous increased bioluminescent signals over time (Fig. 4A). The mice were euthanized at the end of the third week. Tumor tissues were then stripped and weighed. Tumor masses of shSEMA4B-expressing mice were much smaller than shCtrl-expressing ones (Fig. 4B).

### SEMA4B knockdown inhibits the recruitment of MDSCs and T-reg cells

**Fig. 5:**
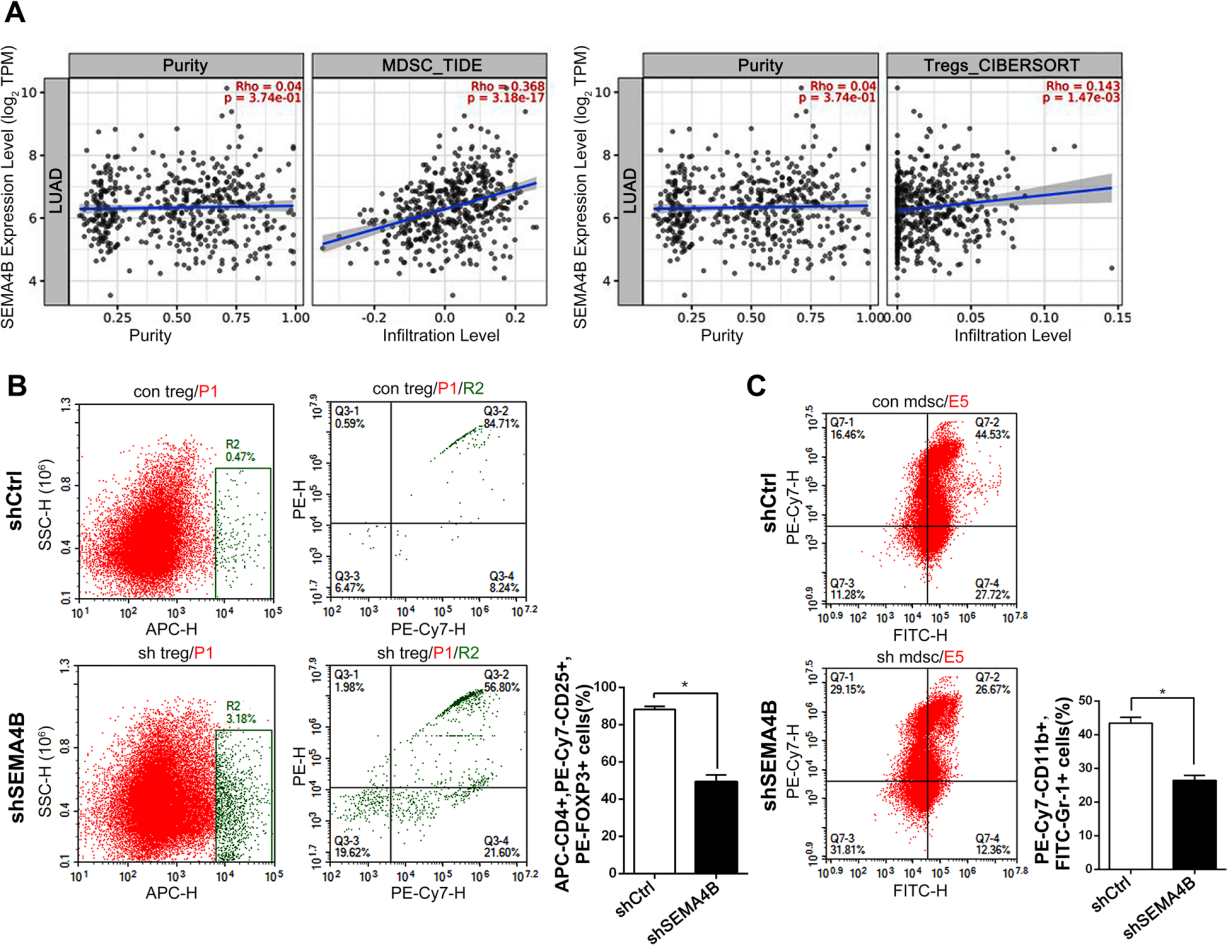
The relation between SEMA4B expression and T-reg cells and MDSCs infiltration in tumor TME. **A** The infiltrates of MDSCs and T-regs in LUAD analyzed by TIMER2.0. **B** The flow cytometry results of CD4+, CD25+, FOXP3 + T-reg cell in shSEMA4B and shCtrl group. **C** The flow cytometry results of CD11b+, Gr-1+ MDSCs

TIMER2.0 database was used to analyze the correlation between SEMA4B expression and immunosuppressive cell infiltration that are known to promote T-cell exclusion in LUAD. As shown in Fig. 5A, SEMA4B expression was significantly positively correlated with tumor infiltration of MDSCs (R = 0.368, *p*<0.001) and Tregs (R = 0.143, *p*<0.05).

For in vivo study, the xenograft tumor tissues of the shSEMA4B and shCtrl group were disaggregated in single-cell suspensions and stained with cell surface markers including CD4, CD25, CD11b, and intracellular markers FOXP_3_ and Gr_1_. The flow cytometry assay suggested that the proportion of CD4^+^, CD25^+^, FOXP_3_^+^ T-reg cells in the shSEMA4B group were significantly lower than the shCtrl group, and SEMA4B downregulation were positively associated with decreased infiltration of CD11b^+^, Gr_1_^+^ MDSCs (Fig. 5B and C), suggesting their interplay with T cell infiltration and tumor progression.

## Discussion

Over 60% of lung cancer patients present with locally advanced or metastatic disease at the time of diagnosis, at which surgical resection may not be an option. In the past decade, significant progresses have been made in lung cancer treatment. Checkpoint inhibitors have been elevated to standard of care in the front-line setting. Nevertheless, the median duration of response was only 18.6 months after single-agent immunotherapy for Chinese patients [24]. It is urgent clinical need to fully elucidate the mechanisms by which immunotherapy exert their efficacy.

Semaphorins, initially identified as axon guidance, play versatile and important roles in regulating multiple hallmarks of cancer including cell proliferation, invasion, metastasis, angiogenesis and tumor-associated inflammation. SEMA4A, 4C and 4D were the three most famous family members found to be involved in the development of multiple malignancies such as breast cancer, colorectal cancer, cervical cancer and ovarian cancer [25]. Recently, a propensity to develop malignant tumors have also been observed in patients with high level of SEMA4B expression. For instance, SEMA4B expression in laryngeal squamous cell carcinoma (LSCC) was significantly upregulated and exogenous transforming SEMA4B confers LSCC cell growth traits [26]. Besides, overexpression of SEMA4B was observed in renal cell carcinoma and contributed to the tumor progression and poor prognosis [27]. In glioma, downregulation of SEMA4B inhibited U87 cell proliferation, clone formation and migration in vitro and attenuated tumorigenicity in vivo [28]. In lung cancer, SEMA4B initially recognized for its ability to stimulate cell motility [29]. Recently, SEMA4B was indicated as an predictor of lymph node metastasis in lung adenocarcinoma [18]. Importantly, bioinformatic study revealed that SEMA4B might act as one of immune system modulators and be related to a shorter survival of NSCLC [19]. However, to our knowledge, the role of SEMA4B in lung cancer immunotherapy has not been thoroughly demonstrated, especially under in vivo conditions.

In the present study, we firstly used RNA-seq data from TCGA database to explore the relation between SEMA4B expression and LUAD features. Our results showed that SEMA4B was highly expressed in LUAD tissues and positively correlated with tumor pathological stage, T and N stage, but not with gender, age and smoking status. To further explore the effect of SEMA4B on the biological behavior of lung cancer, the cell proliferative ability was evaluated. We found that SEMA4B could significantly promote lung cancer cell proliferation both in vivo and in vitro.

Given the limited data on SEMA4B function, we also performed GO, KEGG and GSEA analysis. As shown in sFig. 1, the results indicated that SEMA4B high phenotype was associated with NF-κB activation, chromosome maintenance, cellular senescence, acetylate histones and complement cascade pathway. NF-κB pathway has been previously shown to involve in recruitment, maintenance, and function of MDSCs and Tregs [30]. MDSCs represent a heterogeneous population of immature myeloid cells that are accumulated during tumor progression and exhibit remarkable immunosuppressive and tumorigenic activities by secreting immunosuppressive cytokines such as IL-10 and TGF-β [31]. Tregs play an important role in maintaining immune homeostasis, but they also contribute to tumor immune evasion through competitively binding with receptors on T cells including PD-L1 and CTLA4 [32, 33]. Bioinformatic analysis demonstrated that infiltration of MDSCs and T-regs were positively associated with the expression of SEMA4B, which means SEMA4B might be an immune-related gene in LUAD. Importantly, as shown in the allograft model of Lewis lung cancer, SEMA4B knockdown decreased the enrichment of tumor infiltrating T-regs and MDSCs. We supposed that SEMA4B might mediate tumor immune evasion by stimulating recruitment and infiltration of immunosuppressive cells, and the latter in the TME of LUAD contributed to poor prognosis. Meanwhile, we found SEMA4B expression might be also associated with other tumor-infiltrating immune cells as shown in sfig. 2A. Specifically, it might be negatively correlated with enrichment of CD8^+^ cytotoxic T cell (CTL) and B cell, while positively with infiltration of immunosuppressive TAMs and cancer-associated fibroblast (sFig. 2B). Importantly, discrepancies between prediction results and actual findings about immune cell infiltration pose a challenge. For example, Treg infiltration was supposed to be negatively correlated with SEMA4B expression as shown in sFig. 2A, but there actually existed a positive correlation between them. In future studies, we will focus on the correlation between SEMA4B and the recruitment of CTL, B cells, polarization of TAMs and tumor-associated fibroblasts.

Previous study reported that exogenous expression of SEMA4B inhibited the proliferation and metastasis of A549 cells [34–36], which was inconsistent with our data. The reasons for the paradox are not fully clear. However, some possible reasons might include SEMA4B exhibited a dual role by demonstrating anti-tumor and pro-tumor effects. Furthermore, immunodeficient NOD-SCID mice bearing human-derived A549 tumors were used in their study, while our study selected C57BL/6 mice with intact immune system to establish experimental mouse model in which SEMA4B might regulate fully functional immune cells to exert immune suppression.

## Conclusion

Collectively, we found SEMA4B could promote tumorigenesis through inducing cell proliferation and influencing immune infiltration in the TME of LUAD. High expression of SEMA4B predicts shorter OS of LUAD patients. Although initially being identified as an axon-guidance molecule, SEMA4B has the potential to be a novel prognostic marker to predict treatment outcomes of ICIs treatment. This study provides a new insight for further investigating heterogeneity of immunosuppressive TME in lung cancer.

## Materials and methods

### Pan-cancer analyses

Pan-cancer analyses were performed to compare the expression of SEMA4B in the tumor samples of genotype-tissue expression (GTEx) combined with TCGA. UCSC TOIL was used to correct for batch effects, and to allow for sample merging. In total, 33 different TCGA projects, each representing a specific cancer type, were analyzed. The difference between tumor and normal samples was tested by the Wilcoxon rank sum test.

### SEMA4B differential expression in TCGA LUAD data

Gene expression data from RNA-Seq used in this study were collected from TCGA and GTEx projects (including 515 LUAD tissues and 347 normal tissues). RNAseq data in level 3 HTSeq-FPKM format was converted into transcripts per million (TPM) reads format and was log2 transformed for all downstream analyses. Boxplots and scatter plots were generated to compare differential expression of SEMA4B between tumor or normal tissues. The diagnostic performance of SEMA4B was estimated using receiver operating characteristic (ROC) curves. DESeq2 (3.8) package was used to identify DEGs between SEMA4B-high and SEMA4B-low patients from TCGA datasets [37]. The adjusted. *p* value<0.05 and |log2(fold change)| > 2 were defined as cutoff values for DEGs. All the DEGs were presented in a heat map and a volcano plot.

### Functional enrichment and analysis of immune cell infiltration

GO analysis and KEGG analysis were conducted using R cluster Profiler package (3.14.3) to predict the SEMA4B-related phenotypes and signal pathways [23, 38–40]. An false discovery rate (FDR) q-value < 0.2, fold change≥2, and *p* <  0.05 were considered significant statistically. In GSEA analysis, a permutation test with 1000 times was used to identify the significantly changed pathways. An FDR < 0.25 and adjusted *p* <  0.05 were identified as significant related genes. Statistical analysis and graphical plotting were conducted and visualized using R package ggplot2 (3.3.3) [41]. The tumor infiltration of 24 immune cell types were quantified by single-sample GSEA (ssGSEA) using the R GSVA package based on TCGA [42]. The TIMER2.0 database was then used to analyze the correlation between the expression of SEMA4B and infiltration of CD8+ T cells, B cells, MDSCs, T-regs, cancer associated fibroblast (CAFs) and macrophages. The degree of significance (*p* value) between SEMA4B and immune cell infiltration was < 0.01. A |Spearman’s rank correlation coefficient|>0.2 was considered as positive correlation.

### Evaluation the relationship between SEMA4B expression and LUAD prognosis

The relationship between clinical pathologic features and SEMA4B was analyzed with the Wilcoxon signed-rank sum test and logistic regression. Univariate and multivariate analysis with Cox’s regression model were used to statistically identify the best combination of risk factors to predict prognosis. The OS difference of between patients with high and low SEMA4B expression was calculated by the Kaplan–Meier method with the two-sided log-rank test using two R packages (survival 3.2–10 and survminer 0.4.9). A *p* value<0.05 was considered as significance in all tests.

### Clinical relevance of SEMA4B expression in LUAD patients

A total of 10 human paraffin-embedded tissue samples obtained by surgical resection from LUAD patients were collected from the Department of Thoracic Surgery of Tangdu Hospital. Tissue samples were then investigated for SEMA4B expression by immunohistochemistry (IHC). The rabbit anti-SEMA4B antibody (Sigma, #HPA013372, 1:200) was purchased from Sigma, St. Louis, MO, USA. FOXP_3_^+^ T-reg cells and CD11b^+^ MDSCs were also stained with Rat anti-CD11b (Abcam, ab8878,1:200) and anti-FOXP3 (Abcam, ab215206,1:100) antibody.

### Cell culture and preparation

LLC and HEK293T cells were purchased from Shanghai Institutes for Biological Sciences (Chinese Academy of Sciences, Shanghai, China). The LLC cells were cultured in RPMI 1640 medium with 10% fetal bovine serum (FBS, Biological industries, Beit Ha’emek, Israel), penicillin 100 U/mL and streptomycin 100 U/ml. HEK 293 T cells were cultured in DMEM medium with 10% FBS, penicillin 100 U/mL and streptomycin 100 U/ml. Luciferase plasmid was constructed based on the pLenti6.3 lentiviral vector. Next, HEK293T cells were transfected with luciferase plasmid (4μg) along with the helper plasmid psPAX2 (3μg), and pMD2.G (1μg) using lipofectamine 2000 transfection reagent (Invitrogen, Carlsbad, CA, USA). The viral supernatants were collected in 48 h and 72 h after transfection and used for infection of LLC cells to produce luciferase-labeled LLC cells, which were maintained in 1640 medium supplemented with 10% FBS and 250 μg/ml Hygromycin B (Roche, Switzerland). Lentiviral plasmids encoding shSEMA4B/shCtrl were constructed based on the pLKO.1 vectors. 4 ng of shSEMA4B/shCtrl plasmids was co-transfected into HEK293T cells with helper plasmids using lipofectamine 2000. The viral supernatants were collected in 48 h and 72 h after transfection and used for infection of luciferase-labeled LLC cells with 8 μg/mL polybrene (Sigma, St. Louis, MO, USA). The transduced cells were selected using 2μg/ml Puromycin (Gibco, UK).

### Quantitative real-time PCR

Total RNA was extracted by TRIzol (Invitrogen, Carlsbad, CA, USA) and reverse transcribed into cDNA with RT reagent Kit (Takara Bio Inc. Japan). The difference in expression level of SEMA4B was compared by qPCR (ROCHE lightcycle 96, Indianapolis, IN, USA). Correlation data was calculated based on the ΔΔCT of the target gene and the internal reference actin. SEMA4B-F: 5′-TAGCTTCCAGGGAAACGACC-3′; SEMA4B-R:5′-CCCGTCTCGCTGAAGAAGAA-3′. hACTIN-F: 5′-CGGCACCACCATGTACCCTG-3′;hACTIN-R: 5′- ACACGGAGTACTTGCGCTCA′-3′.

### Cell proliferation assay

Cells proliferation capacity was evaluated with CCK-8 and Edu assay. Firstly, 5 × 10^3^ cells/well LLC cells expressing shSEMA4B or shCtrl were seeded at 96-well plates, and cultured at 37 °C. The viability of the cells was detected according to the manufacturer’s instructions. 10 μL Cell Counting Kit-8 (CCK-8; Dojindo Laboratories, Kumamoto, Japan) was added at the indicated time points and cells were incubated for 2 h at 37 °C. Finally, the absorbance of each well at 450 nm was measured with a microplate reader (Tecan, Männedorf, Switzerland).

EdU staining was conducted according to the manufacturer’s instruction (US everbright inc, Suzhou, China). Briefly, 1 × 10^4^ LLC cells expressing shSEMA4B or shCtrl were seeded at 96-well plate, and (3 sub-wells were set up for each group). After incubated at 37 °C for 24 hours, each well was added with 100 μl of solution A with a final concentration at 5%. 4 hours later, cells were fixed with 4% paraformaldehyde for 15 min, and then neutralized with 100 μl of glycine (2 mg/ml) for 5 min, and an addition of 100 μl 0.5% TritonX-100 for 20 min. 80 μl of C solution (1×), 4 μl of D solution, 0.2 μl of B solution, 10 μl E (1×) was added to each well. After being stained in darkness for 30 min at room temperature, cells were incubated with 100 μl of 1% F solution for 30 min, and counted under a fluorescence microscope.

### Plate clone formation assay

Briefly, cells expressing shSEMA4B or shCtrl were seeded in 6-well plates at a density of 10^3^ cells/well for 7–14 days and then fixed with 4% paraformaldehyde for 20 min. After being stained with 0.1% crystal violet for 30 min, cells were rinsed and scanned to grayscale image by Odyssey infrared imaging system (LICOR, USA).

### Animal experiments

5-week-old of female C57BL/6 mice were purchased from the Animal Experimental Center of the Fourth Military Medical University. 2 × 10^6^ LLC tumor cells expressing shSEMA4B or shCtrl were implanted subcutaneously in the thigh of C57BL/6 mice. 2 to 3 weeks later, the tumor masses maintained in mice were observed after being intraperitoneally injected with 150 mg/kg Dluciferin. Living images were captured by an IVIS imaging system (PerkinElmer, life sciences, USA). All the experimental procedures involving animals were conducted under a protocol reviewed and approved by the Ethics Committee of Tangdu Hospital, Fourth Military Medical University.

### Flow cytometry and immunohistochemistry assay

After being harvested and weighed, tumor tissues were cut into small pieces and mixed with IV collagenase for 2 h. Tissues were homogenized and filtered through a 70 μm filter to remove remaining undigested tissue. The single cell suspension was centrifuged at 500 g for 5 min, incubated with 2 μl extracellular antibody for 30 min, and then fixed with 4% paraformaldehyde for 20 min. Finally, the suspension was incubated with intracellular antibody for another 30 min before being tested by flow cytometry (NovoCyte, ACEA Biosciences, San Diego, USA). APC-Anti-CD4 (#553051), PE-Cy7-anti-CD25(#552880), PE-anti-FOXP3(#563101), PE-Cy7-anti-CD11b (#552850) and FITC-anti-Gr-1(#553126) were purchased from BD pharmingen™.

### Statistical analysis

All experiments were performed in triplicate on three independent occasions. Data are expressed as mean ± S.D. One-way ANOVA was employed for statistical analysis by SPSS 22.0 (SPSS, Chicago, USA). The differences between means were tested by an independent sample *t-test*. The association between SEMA4B expression and clinico-pathological parameters was examined by *χ2* test. A *p* value < 0.05 was considered significant. * *p* < 0.05, ** *p* < 0.01, *** *p* < 0.001**.**

## Supplementary Information

**Additional file 1: Supplementary Fig. 1.** Significantly enriched GO and SEMA4B-related pathways in LUAD. (A) BP (biological process), CC (cellular component), MF (molecular function) and KEGG enrichment related to HTRA3 related genes with bubble chart. (B) Enrichment plots from the gene set enrichment analysis (GSEA). Several pathways and biological processes were differentially enriched in SEMA4B-related GC, including activated NF-kB activation, chromosome maintenance, cellular senescence, histone acetyltransferases (HATs) and complement cascade pathway. NES = normalized enrichment score; p.adj = adjusted *P* value; FDR = false discovery rate.

**Additional file 2: Supplementary Fig. 2.** Correlation of immune cell infiltration and SEMA4B expression in LUAD patients. (A) Relationships among infiltration levels of 24 immune cell types and SEMA4B expression profiled by Spearman’s analysis. (B) Shown is the relation between SEMA4B expression and immune cell infiltration in tumor TME analyzed by Timer2.0 database, including CD8+ T cell, cancer associated fibroblast, B cell and macrophages. DCs, dendritic cells; aDCs, activated DCs; iDCs, immature DCs; pDCs, plasmacytoid DCs; Th, T helper cells; Th1, type 1 Th cells; Th2, type 2 Th cells; Th17, type 17 Th cells; Treg, regulatory T cells; Tgd, T gamma delta; Tcm, T central memory; Tem, T effector memory; Tfh, T follicular helper; NK, natural killer.

**Additional file 3: Supplementary Fig. 3.** The staining of CD11b and Foxp3, which represented for MDSCs and Tregs in normal and LUAD specimens, respectively. (100 × , bar = 200 μm).

## Data Availability

The original contributions presented in the study are included in the article/Supplementary Material. Further inquiries can be directed to the corresponding author.
