# Supplementary figures and images for: Semaphorin 4B promotes tumor progression and associates with immune infiltrates in lung adenocarcinoma
Source: BMC Cancer. 2022 Jun 8;22:632. doi: 10.1186/s12885-022-09696-w (PMC9178879; doi:10.1186/s12885-022-09696-w)

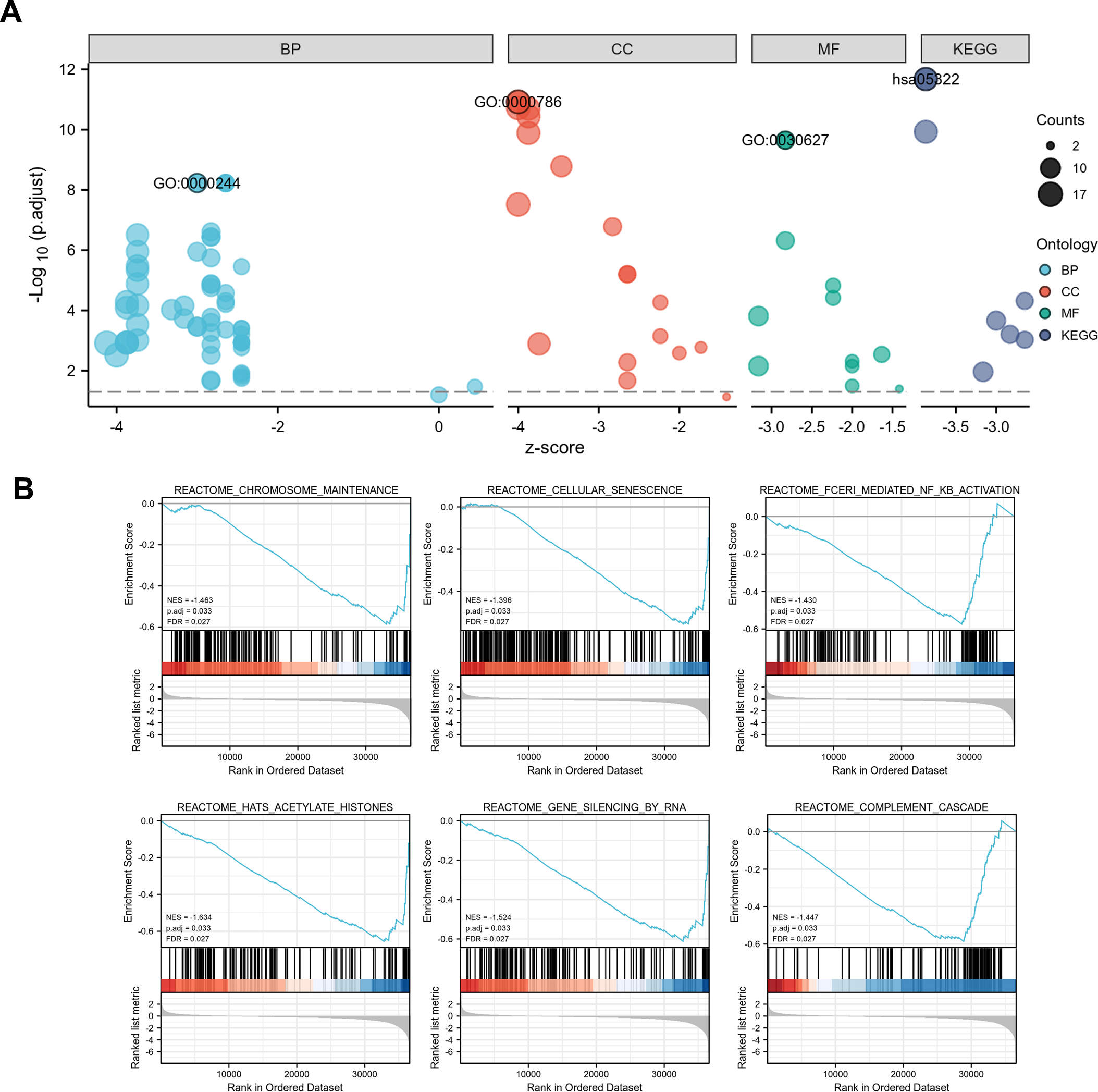

Supplement: Supplementary file 1 — Additional file 1: Supplementary Fig. 1. Significantly enriched GO and SEMA4B-related pathways in LUAD. (A) BP (biological process), CC (cellular component), MF (molecular function) and KEGG enrichment related to HTRA3 related genes with bubble chart. (B) Enrichment plots from the gene set enrichment analysis (GSEA). Several pathways and biological processes were differentially enriched in SEMA4B-related GC, including activated NF-kB activation, chromosome maintenance, cellular senescence, histone acetyltransferases (HATs) and complement cascade pathway. NES = normalized enrichment score; p.adj = adjusted P value; FDR = false discovery rate. [file 12885_2022_9696_MOESM1_ESM.tif]

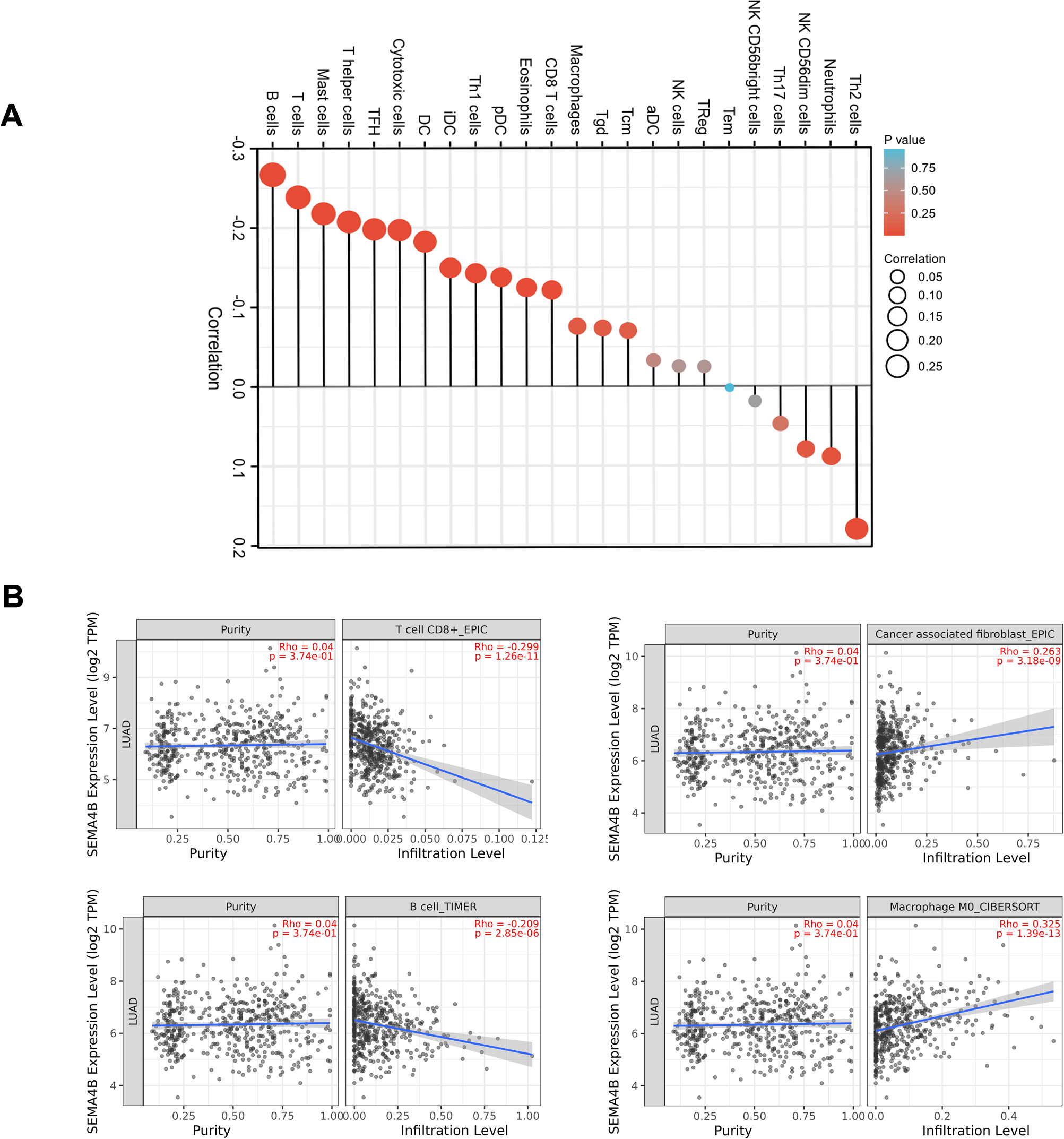

Supplement: Supplementary file 2 — Additional file 2: Supplementary Fig. 2. Correlation of immune cell infiltration and SEMA4B expression in LUAD patients. (A) Relationships among infiltration levels of 24 immune cell types and SEMA4B expression profiled by Spearman’s analysis. (B) Shown is the relation between SEMA4B expression and immune cell infiltration in tumor TME analyzed by Timer2.0 database, including CD8+ T cell, cancer associated fibroblast, B cell and macrophages. DCs, dendritic cells; aDCs, activated DCs; iDCs, immature DCs; pDCs, plasmacytoid DCs; Th, T helper cells; Th1, type 1 Th cells; Th2, type 2 Th cells; Th17, type 17 Th cells; Treg, regulatory T cells; Tgd, T gamma delta; Tcm, T central memory; Tem, T effector memory; Tfh, T follicular helper; NK, natural killer. [file 12885_2022_9696_MOESM2_ESM.tif]

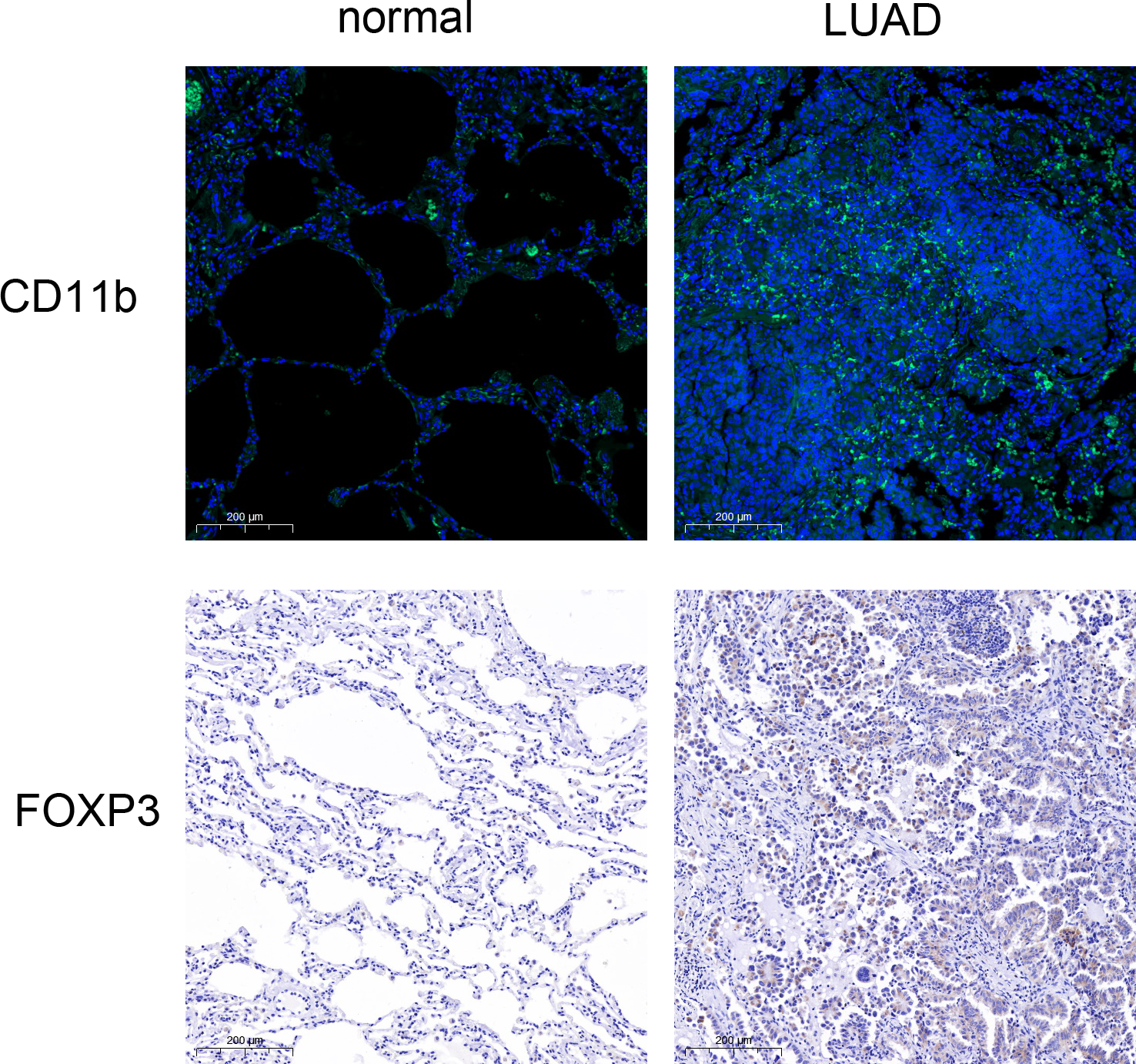

Supplement: Supplementary file 3 — Additional file 3: Supplementary Fig. 3. The staining of CD11b and Foxp3, which represented for MDSCs and Tregs in normal and LUAD specimens, respectively. (100 × , bar = 200 μm). [file 12885_2022_9696_MOESM3_ESM.tif]
